# Widespread horizontal genomic exchange does not erode species barriers among sympatric ducks

**DOI:** 10.1186/1471-2148-12-45

**Published:** 2012-04-02

**Authors:** Robert HS Kraus, Hindrik HD Kerstens, Pim van Hooft, Hendrik-Jan Megens, Johan Elmberg, Arseny Tsvey, Dmitry Sartakov, Sergej A Soloviev, Richard PMA Crooijmans, Martien AM Groenen, Ronald C Ydenberg, Herbert HT Prins

**Affiliations:** 1 Resource Ecology Group, Wageningen University, P.O. Box 47, 6700 AA Wageningen, The Netherlands; 2 Conservation Genetics Group, Senckenberg Research Institute and Natural History Museum, D-63571 Gelnhausen, Germany; 3 Department of Molecular Biology, Faculty of Science, Nijmegen Centre for Molecular Life Sciences, Radboud University Nijmegen, Nijmegen, The Netherlands; 4 Animal Breeding and Genomics Centre, Wageningen University, Marijkeweg 40, 6709 PG Wageningen, The Netherlands; 5 Aquatic Biology and Chemistry, Kristianstad University, SE-291 88 Kristianstad, Sweden; 6 Biological Station Rybachy of the Zoological institute RAS, 238535 Kaliningrad region, Russia; 7 Ecological Watch of Siberia, Komarova street 27/6/5, 644074 Omsk, Russia; 8 Department of Chemistry, Omsk State University, St. Prospect Mira 55a, 644077 Omsk, Russia; 9 Centre for Wildlife Ecology, Simon Fraser University, V5A 1S6 Burnaby, BC, Canada

## Abstract

**Background:**

The study of speciation and maintenance of species barriers is at the core of evolutionary biology. During speciation the genome of one population becomes separated from other populations of the same species, which may lead to genomic incompatibility with time. This separation is complete when no fertile offspring is produced from inter-population matings, which is the basis of the biological species concept. Birds, in particular ducks, are recognised as a challenging and illustrative group of higher vertebrates for speciation studies. There are many sympatric and ecologically similar duck species, among which fertile hybrids occur relatively frequently in nature, yet these species remain distinct.

**Results:**

We show that the degree of shared single nucleotide polymorphisms (SNPs) between five species of dabbling ducks (genus *Anas*) is an order of magnitude higher than that previously reported between any pair of eukaryotic species with comparable evolutionary distances. We demonstrate that hybridisation has led to sustained exchange of genetic material between duck species on an evolutionary time scale without disintegrating species boundaries. Even though behavioural, genetic and ecological factors uphold species boundaries in ducks, we detect opposing forces allowing for viable interspecific hybrids, with long-term evolutionary implications. Based on the superspecies concept we here introduce the novel term "supra-population" to explain the persistence of SNPs identical by descent within the studied ducks despite their history as distinct species dating back millions of years.

**Conclusions:**

By reviewing evidence from speciation theory, palaeogeography and palaeontology we propose a fundamentally new model of speciation to accommodate our genetic findings in dabbling ducks. This model, we argue, may also shed light on longstanding unresolved general speciation and hybridisation patterns in higher organisms, e.g. in other bird groups with unusually high hybridisation rates. Observed parallels to horizontal gene transfer in bacteria facilitate the understanding of why ducks have been such an evolutionarily successful group of animals. There is large evolutionary potential in the ability to exchange genes among species and the resulting dramatic increase of effective population size to counter selective constraints.

## Background

Biology has seen the proposition of several species concepts. Of these, the biological species concept [1] is historically the most influential; according to it all individuals belong to the same species if they produce viable and fertile offspring in nature, i.e., they share a common gene pool. To account for inherent difficulties to test this concept in practice, especially in allopatric populations that never encounter each other, biologists tend to supplement it by elements of the morphospecies concept (which is as old as the study of nature). With the advance of molecular genetic data over the past decades many researchers now define species by genetic characteristics rather than morphological ones because genetics provides a means of actually measuring recent or ongoing genetic connectivity between species [2]. Species boundaries are strengthened by accumulation of genomic incompatibilities preventing formation of zygotes, so called Dobzhansky-Muller incompatibilities [3-5]. Once evolved, post-zygotic isolation is irreversible, in contrast to pre-zygotic barriers such as mate recognition. There is much evidence that post-zygotic barriers evolve slowly in birds [5,6], potentially contributing to the high rates of hybridisation observed in this group [7] and explaining why genetic distances can be low in spite of large morphological differences [8].

When populations diverge into species their gene pools become disconnected, and even in the absence of ecological differentiation stochastic effects, i.e., genetic drift, will drive each new species towards increased differentiation. If introgression of genetic material of one species into another occurs regularly enough in the absence of genomic incompatibility, one would expect that these events oppose genetic drift by exchange of alleles that the two subsequently will have in common. Such potential sharing of alleles at genetic loci through genetic admixture can directly be observed by the study of genetic markers. One type of genetic marker that has recently received a lot of attention is the 'single nucleotide polymorphism' (SNP) [9]. Due to the abundance of SNPs in genomes and suitability for high automation in genotyping, SNPs can be characterised in large numbers, yielding a representative image of an entire genome. With SNP data from multiple species, one can study the sharing of genetic material at the same loci, providing a new means of studying species divergence by the speed of loss of genetic coherence.

**Figure 1:**
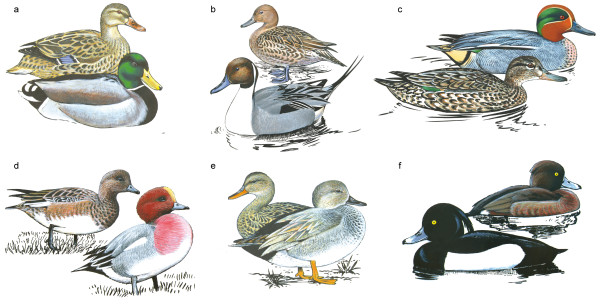
**The studied duck species**. Male and female of each of the studied duck species: a) *Anas platyrhynchos *(mallard), b) *Anas acuta *(northern pintail), c) *Anas crecca *(teal), d) *Anas penelope *(Eurasian wigeon), e) *Anas strepera *(gadwall), f) *Aythya fuligula *(tufted duck). Except for c) the more colourful male is in the front. Evidently, plumage ornamentation in these ducks is very distinctive among species. Drawings are from the artwork stocks of the WWT, Slimbridge, UK, and used with permission.

While persistent genetic admixture can lead to the merging of species [10,11] this does not generally seem to be the case in some taxonomic groups. For example, ducks (family Anatidae) show much hybridisation in the wild, with viable and fertile offspring [7,12,13]. In spite of this, duck species remain morphologically distinct. Males especially display species-specific plumage, ornamentation, and courtship behaviour (Figure 1). In the present study, we utilise a recently developed SNP set for the mallard (*Anas platyrhynchos*) [14] to infer the degree of genomic connectivity among five species of closely related, ecologically similar and morphologically well differentiated duck species, among which interspecific hybridisation is commonplace. With this example we set out to illustrate how analysis of "SNP persistence time" facilitates the understanding of the evolutionary impact of ongoing hybridisation, how it can reveal the existence of superspecies complexes, and how it sheds light on longstanding unresolved puzzles of speciation processes.

## Results and Discussion

### Genotypic differentiation between *Anas platyrhynchos *and other duck species

We screened 364 SNPs developed for the mallard, *Anas platyrhynchos*, [14] in the genomes of six duck species, five of genus *Anas *and one of *Aythya*, the latter mainly for outgroup comparison: *Anas platyrhynchos *(*N *= 197), *Anas acuta *(northern pintail, *N *= 7), *Anas crecca*, (common teal, *N *= 9), *Anas penelope *(Eurasian wigeon, *N *= 14), *Anas strepera *(gadwall, *N *= 10) and *Aythya fuligula *(tufted duck, *N *= 17). The SNPs were evaluated for minor allele frequency (MAF) spectrum, Hardy-Weinberg equilibrium and linkage disequilibrium in *Anas platyrhynchos *from nine localities on three continents. The great majority of SNPs does not significantly deviate from neutrality and are unlinked.

**Figure 2:**
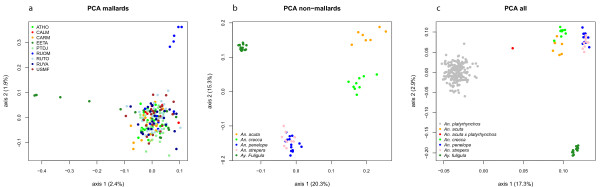
**PCA analysis of duck genotypes**. The program smartpca from the Eigenstrat package was used to calculate multivariate eigenvectors of the duck genotypes. The first two eigenvectors for each individual are plotted and colour coded by locality or species. The percent variation explained by PCA axes 1 and 2 is given in brackets. a) only* Anas platyrhynchos *individuals, colour coded by locality (see additional file 4). b) other ducks, colour coded by species: *An. acuta *(*Anas acuta*, ANAC), *An. crecca *(*Anas crecca*, ANCR), *An. penelope *(*Anas penelope*, ANPE), *An. strepera *(*Anas strepera*, ANST), *Ay. fuligula *(*Aythya fuligula*, AYFU). c) A joint calculation of PCA axes including all ducks analysed in this study. Additionally, a hybrid between *Anas acuta *and *Anas platyrhynchos *was included (ANACPLA), which is placed between the *Anas platyrhynchos *and *Anas acuta *cluster as expected. *Anas platyrhynchos *clearly forms an own cluster and the genetic similarity to the other species clusters reflects phylogenetic placements (i.e., *Anas platyrhynchos *is more closely related to *Anas acuta *and *Anas crecca *than to *Anas penelope *or *Anas strepera*).

We plotted the results of a series of principal component analyses (PCAs) for several combinations of individual of *Anas platyrhynchos *and other species genotypes. All plots are based on the first and second PCA axes. Other axes were investigated visually but did not provide further insight. No clear genetic clusters among specimens of *Anas platyrhynchos *were discernible in this analysis when analysed separately, and the evident absence of genetic structure in mallards is reflected by low values of explained variance in the first and second PCAs (Figure 2a). Geography had no influence on genetic similarity. Even after correcting for potential mislabelling or outliers (see methods for details) a few individuals seem to lie a bit outside the main cluster, but note that the scaling of differences between *Anas platyrhynchos *individuals in this PCA is different from the scaling in analyses involving other duck species (see below). Interestingly, a lack of population structure in mallards has also been described on a continent-scale for mitochondrial data [15] and on a global scale using SNPs (Kraus *et al.*, manuscript submitted). The other species form distinct clusters if analysed together (Figure 2b): *Anas penelope *and *Anas strepera *form one cluster and are hard to distinguish from each other. *Anas acuta *and *Anas crecca *each form their own specific clusters. *Aythya fuligula *is of a different genus and hence not a dabbling duck. It serves as outgroup here and clearly lies outside these clusters. When individuals of all species are analysed jointly in this way (Figure 2c), *Anas platyrhynchos *is clearly distinct from the other species. A putative hybrid between *Anas acuta *and *Anas platyrhynchos *is placed exactly in between its assumed parental species, thereby confirming its supposed hybrid status.

### SNP sharing among duck species is unexpectedly high

**Figure 3:**
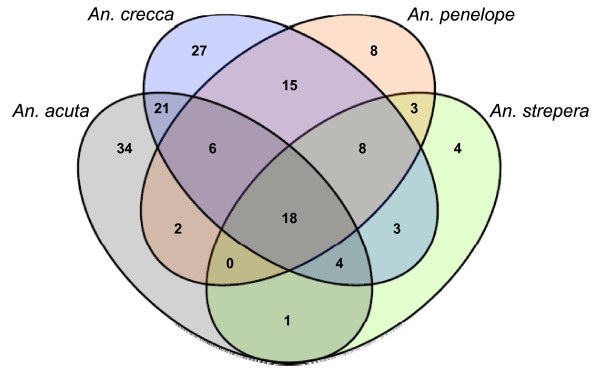
**Venn diagram of shared SNPs with mallard by the four other *Anas *species**. A core of 18 SNPs was polymorphic in all four *Anas *species. The closer phylogenetic relationship of *Anas acuta *and *Anas crecca *to *Anas platyrhynchos *is reflected in their polymorphism sharing pattern. Abbreviations as in Figure 2.

**Figure 4:**
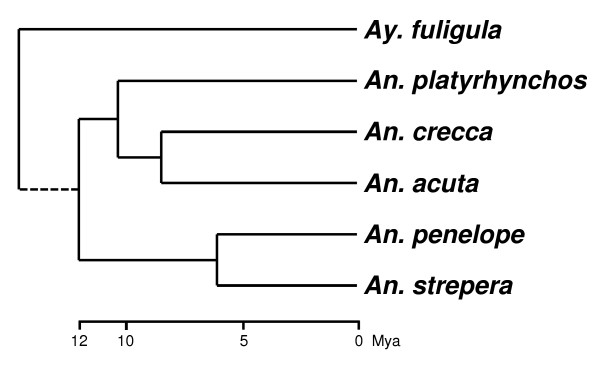
**Schematic phylogram of the studied duck species**. Branch lengths are scaled to Mya. *Aythya fuligula *was added as outgroup (branch length shortened at the split of the genus). Redrawn from [22] and Javier Gonzales (pers. comm.). *An*. codes for the genus *Anas*, and *Ay*. for* Aythya*.

Genotyping was successful in the non-*Anas platyrhynchos *species with only 14-24% missing genotypes while within *Anas platyrhynchos *(for which the SNP set was originally designed) this number was 4%. Of 364 *Anas platyrhynchos *SNPs, 86 (24%) were polymorphic in *Anas acuta*, 102 (28%) in *Anas crecca*, 60 (16%) in *Anas penelope*, 41 (11%) in *Anas strepera*, and 11 (3%) in *Aythya fuligula *(Figure 3). The proportion of shared SNPs between the *Anas *species are high compared with those reported in studies comparing other species with similar evolutionary distances. Bovines (cattle, bison and yak), for instance, have a relatively recent, Pleistocene radiation 2.5 million years ago (Mya), yet SNP sharing does not exceed 5% [16]. SNP sharing in the genus *Gallus *(chickens and relatives), another taxon with putative Pleistocene speciation and recent introgression from domestic animals, is also estimated at 5%[17], while in sheep (divergence time ~ 3 Mya) it is estimated at only 1% [18]. The same low levels of SNP sharing also occur in invertebrate and plant species. The flies *Drosophila pseudoobscura *and *D. miranda *show 2.9% SNP sharing [19] (divergence time 3.7 Mya [20]) while the plant pairs *Arabidopsis halleri*/*A. lyrata petraea *and *A. lyrata lyrata*/*A. l. petraea *share 4.7% and 1.6%, respectively [21] (divergence times < 5 Mya). Given the divergence time of *Anas platyrhynchos *from, e.g., *Anas acuta *and *Anas crecca *of at least 6.4 Mya [22] (Figure 4) they share up to an order of magnitude more SNPs than shown in these previous reports.

Generally, the rate of SNP sharing in closely related species, as reported thus far, appears to be in the order of a few percent, at maximum. Random genetic drift usually purges polymorphisms as a function of time (generations), effective population size (N_e_) and initial MAF, allowing an approximation of the time to fixation of allele frequencies under genetically neutral conditions [23]. For *Anas platyrhynchos *we estimate the mean persistence time (i.e., how long the polymorphisms segregate) for alleles with the highest possible MAF to be 5.3 million years, assuming a generation time of one year and N_e _being constant at the present-day number. In the other duck species studied here it ranges between 0.8 and 2 million years. Rare alleles, e.g. MAF < 0.1, are lost more quickly (Table 1). The probability distribution for this loss has a long tail towards longer persistence times, with 5% of the shared polymorphisms with a MAF = 0.5 expected to be retained after a calculated threshold of 3.8N_e _generations [24]. For *Anas platyrhynchos *this would equate to 7.2 million years (at a divergence from *Anas crecca*/*Anas acuta *of 6.4 Mya [22]). Thus, *Anas platyrhynchos *could have retained some of the ancestral shared polymorphisms since that split. However, *Anas acuta *and *Anas crecca *currently have much smaller N_e_, and are unlikely to have retained more than 5% of their ancestral polymorphisms for periods longer than 2 and 2.6 million years (on the basis of 3.8N_e _generations), if these species were reproductively fully isolated. Even with three times higher N_e _or generation time, the number of shared SNPs between the studied duck species is higher than expected: the persistence times of the 5% fraction of SNPs with MAF = 0.5 for *Anas acuta *and *Anas crecca *(6.2 and 7.9 Mya) just exceed their divergence time from *Anas platyrhynchos *(6.4 Mya [22]). On the other hand, under these scenarios *Anas penelope *and *Anas strepera *would not have retained more than 5% of SNPs with MAF = 0.5 after 3.8 and 4.3 million years, respectively, at a minimum divergence time from *Anas platyrhynchos *of 8 Mya [22]. In conclusion, it seems the number of shared SNPs between the studied duck species exceeds what is likely under the neutral theory even when conservatively high estimates of N_e _(from the upper bounds of the official counts) and conservatively low divergence times (mean times minus standard deviation of the values presented in [22]) are assumed.

**Table 1:** Interaction between population size and persistence time

	Census size N_c_	Effective size N_e_	Persistence time (mean)	
			p = 0.5	p = 0.1

*Anasplatyrhynchos*	19,000,000	1,900,000	5,267,919	2,470,631

*Anasacuta*	5,400,000	540,000	1,497,198	702,179

*Anascrecca*	6,900,000	690,000	1,913,086	897,229

*Anaspenelope*	3,300,000	330,000	914,954	429,110

*Anasstrepera*	3,800,000	380,000	1,053,584	702,179

*Aythyafuligula*	2,900,000	290,000	804,051	377,096

Population sizes N_c _and N_e _and mean persistence times in generations of the most balanced (*p *= 0.5; maximum frequency) and a rare (p = 0.1) SNP in each duck species. A ratio of 0.1 for N_e_/N_c _was assumed (see methods for more info).

### Increased population size by ongoing interspecific hybridisation

What can then explain the high level of shared polymorphisms? We argue that these (and other closely related) duck species are part of a superspecies complex, here defined as a group of distinct species that frequently hybridise, with fertile offspring as the result. The superspecies concept was put forward by Mayr in 1931 [25], as a translation of the German expression *Artenkreis*, based on the work of Rensch [26]. Initially, it was used to assign species status to allopatric "races" that were too distinct to be lumped into the same species [27-29] (superspecies *sensu stricto*). Later, the definition was widened by Kiriakoff [30] and Mayr and Short [31] to be no longer exclusive to allopatric populations. For the *Anas platyrhynchos *complex this concept has previously been used by Scherer [13]. Being aware that "superspecies" is not an official taxonomic category we here choose to use the term superspecies (*sensu lato*) to embrace the sympatric distribution of interbreeding duck species. In doing so, we do not attempt to redefine nomenclatural classification schemes, nor do we propose to change current nomenclature. The term superspecies is clearly "an evolutionary taxonomy category but not nomenclatural rank" [32], thus to be preferred when studying biological systems rather than nomenclature.

There is longstanding anecdotal, morphological and experimental evidence for high hybridisation rates in ducks [7,12,22], but molecular proof has been limited thus far. Two studies using mitochondrial DNA in the *Anas rubripes*/*platyrhynchos *[33] and *Anas zonorhyncha*/*platyrhynchos *[34] complexes confirm hybridisation between these species. These findings were corroborated by studies investigating one to two nuclear markers [35,36]. Our study, using shared polymorphisms at hundreds of independent loci across the entire genome provides a more powerful means of analysing gene pool connectivity between closely related species and our results are consistent with a high level of genetic transfer between species via hybrid production and backcrossing.

A STRUCTURE [37] analysis identified several cases where genetic admixture from other species seems supported by their genotypes. When all six duck species were analysed jointly with the genetic clustering software STRUCTURE, all non-*Anas platyrhynchos *individuals were assigned to the same cluster (Additional file 1). *Anas acuta *individuals in particular showed partial *Anas platyrhynchos *genome admixture, and many *Anas platyrhynchos *individuals displayed some admixture from other species. When *Anas platyrhynchos *individuals were excluded, STRUCTURE assigned *Anas penelope*, *Anas strepera *and *Aythya fuligula *individuals to their species specific clusters, although one *Anas strepera *individual (ANST001) was almost fully assigned to *Anas penelope. Anas acuta *and *Anas crecca *were lumped into one cluster, and the hybrid was correctly assigned to that cluster by only 50% of its genome (Additional file 2). Excluding the hybrid from analysis did not alter the assignment of these two species to the same cluster. The same data sets were analysed with comparable settings in the software InStruct [38], which does not assume Hardy-Weinberg equilibrium in the inferred populations, and yields qualitatively similar results as the STRUCTURE analysis. This may be direct evidence of partial gene pool sharing between species, hence the establishment of a superspecies complex.

For example, a superspecies complex comprising *Anas platyrhynchos*, *Anas acuta *and *Anas crecca *would have a joint census population size of 31 million individuals and hence an N_e _of 3.1 million (see methods for sources and assumptions), although sub-division of this possible superspecies due to assortative mating makes this an over-estimate[39]. However, an N_e _of 3.1 million results in a mean persistence time of almost 9 million years (for initial MAF = 0.5). With an estimated most recent common ancestor at 6.4 Mya, these species could have on average retained even SNPs of lower MAF = 0.2. We refer to this analysis as 'persistence time analysis'.

### Species status and the supra-population concept

The ducks studied here have not only remained morphologically distinct, their genetic cluster species designation [2] is strongly supported by principal component analysis of SNP genotypes: we find clear genetic differentiation between *Anas platyrhynchos *and the other duck species, as well as among these (Figure 2c). Even though all these species live in sympatry, such a combined population is highly structured by assortative mating. While geographical substructure would be indicated by the term "meta-population", the situation in ducks leads us to define a new term that does not have a geographical connotation: "supra-population". We define a supra-population as a group of individuals that are part of the same sympatric superspecies complex and within which natural hybridisation occurs. Individuals of a superspecies complex are genetically-connected hybridising species, in which species barriers are primarily maintained by pre-zygotic factors.

### A new model of speciation in ducks

Genomic incompatibilities usually lead to irreversible post-zygotic isolation of populations, but other, reversible, barriers can also be strong drivers of speciation. Visual cues have been identified as drivers of speciation in sexually dimorphic bird species [8,40] while sexual imprinting alone can explain assortative mating in modeling studies [41]. An empirical example from another Anatid species, the snow goose *Anser caerulescens*, which has two wide ranging colour morphs, nicely illustrates the case [42]. At any rate, a model for speciation in ducks must be able to explain the observed pattern of genetic and morphological differentiation in spite of the high degree horizontal gene exchange.

Paleogeographic and paleoclimatic evidence suggest that ecological conditions have been favourable for a duck radiation 6-12 Mya. This late Miocene period was warm and humid [43,44], but in transition towards a colder climate. Precipitation remained relatively high [45-47], making wetlands abundant and turning large inland salt water bodies brackish or even freshwater (e.g., Lake Pannon in Eurasia [48-50]). Globally, during this transition towards a colder, wet climate tropical forests were largely replaced by open grasslands [51-53], a habitat well suited for ducks. The fossil record of ducks beyond the Pleistocene is still very poor [54] but the few studies on the subject suggest that morphological change in respective duck species has been very limited over the last few million years [55,56], after a larger waterfowl species turn-over 15-23 million years ago [57]. The first fossil that resembles *Anas platyrhynchos *is thought to be from the late Pliocene, about 5 Mya [58]. This is close to the suggested lower bound of divergence times of some *Anas *species in the latest phylogeny of Anatidae [22]. We propose that an *Anas*-like duck split into multiple sister morphs sympatrically and simultaneously at that time, subsequently diverging by assortative mating. Our results indicate that the resulting cluster of species still exchanges portions of their genomes. We argue that since branching off of the *Anas *clade at least 6 Mya these mostly sympatric species remain separate by isolating mechanisms other than genetic incompatibilities, mostly by assortative mating. Though we acknowledge that this speciation scenario rests on the assumption of widespread sympatry for millions of years, we feel comfortable in making this claim. Although we only sampled five species for the present study, our model system *sensu lato *is the specious genus *Anas*, and even though species distributions change over time there certainly have always been several *Anas *species living in sympatry.

Theoretical studies suggest that sexual imprinting can drive speciation even in sympatry [59]. Moreover, experimental manipulations clearly demonstrate that individuals of *Anas platyrhynchos *can be imprinted on nearly any species of waterfowl but when raised in isolation they recognise conspecifics as mates [60]. This suggests that imprinting is important but incomplete in ducks; genetic factors also contribute to mate recognition. The presence of assortative mating and recognition mechanisms are prerequisites for sympatric speciation leading to a superspecies complex around *Anas platyrhynchos*.

## Conclusions

The amount of shared polymorphism between the studied duck species cannot be explained by large population sizes of the respective species only. We suggest extraordinary and evolutionarily sustained hybridisation rates as drivers of ongoing gene pool mixing. Gene flow continues and will allow the transfer of genetic material among duck species. At present, extensive hybridisation still occurs. The genetic compatibility of different duck species, combined with mixed effects of genetically determined and imprinted mate choice leads to speciation reversals [11] despite genotypically and morphologically defined species boundaries. Present-day occurrence of *Anas platyrhynchos *in large numbers and wide geographical extent may even drive some of their close relatives to extinction by hybridisation [61]. This is a major concern in many parts of the world, especially where *Anas platyrhynchos *is not indigenous [62]. Many species of the genus *Anas *are hard to fit into the biological species concept because their evolution has rather led to a superspecies complex with discernable lineages. Besides the five dabbling duck species studied here, it is likely that many more of the ca. 40 *Anas *species are part of the global supra-population.

Besides conservation implications, this creates large evolutionary potential, comparable to bacteria, which are able to exchange genes among different species by horizontal gene transfer. Further, increasing effective population sizes into the millions may allow non-adaptive evolutionary processes to act, opening up additional degrees of evolutionary freedom [63]. SNP-based analysis at hundreds of independent loci across the entire genome, as done here, may serve to re-evaluate long-standing puzzling patterns of speciation and hybridisation in several bird groups, such as other waterfowl, galliforms, hummingbirds and woodpeckers [7], as well as in many other organisms where species pairs exhibit unusually high levels of hybridisation.

## Methods

### Samples

In total, 212 individuals of *Anas platyrhynchos *obtained from nine localities representing Eurasian and North American populations were sampled and their blood was stored on FTA cards [64] at room temperature until DNA isolation. Numbers of samples with abbreviation codes in brackets: Eurasian samples were from Austria (25, ATHO), Estonia (22, EETA), Portugal (32, PTDJ), and three Russian localities: Yaroslavl (25, RUYA), Omsk (12, RUOM) and Tomsk (32, RUTO). North America was represented by Ontario (7, CALM), Manitoba (20, CARM) and Alaska (22, USMF). Preliminary multivariate clustering of SNP genotypes (see below) positioned 15 of these individuals far outside the *Anas platyrhynchos *species cluster, sometimes well within the clusters of other duck species (Additional file 3). We discarded these 15 individuals as mislabelled because they showed obvious deviation from their putative genotypic species cluster. Details are available in Additional file 4.

A set of 67 samples from other duck species were obtained world wide from various sources (hunting bags, live-trapped, zoos) and localities. Most often blood on FTA cards was used, sometimes other tissues stored in ethanol, and also previously isolated DNA from collections. The cross species testing was applied to ducks of the following *Anas *and *Aythya *species (numbers of samples and abbreviation code in brackets): *Anas acuta *(7; ANAC), *Anas crecca *(9; ANCR), *Anas penelope *(14; ANPE), *Anas strepera *(10; ANST), *Aythya fuligula *(17; AYFU) and one F_1 _hybrid between *Anas acuta *and *Anas platyrhynchos *(ANACPLA). Using the same procedure as with the *Anas platyrhynchos *set, we identified nine of these samples as apparently mislabelled. These were excluded from all subsequent analyses (Additional file 5). Details are available in Additional file 6.

### DNA isolation

DNA isolation was done using the Gentra Systems Puregene DNA purification Kit according to the manufacturer's instructions, with modifications when handling of FTA cards. Appropriate amounts of tissue or blood on FTA cards were digested with 9 μg Proteinase K (Sigma) in Cell Lysis Solution (Gentra Systems) at 65°C over night, or longer in case of some tissues. Proteins were subsequently precipitated with Protein Precipitation Solution (Gentra Systems) and spun down together with the FTA card material. DNA from the supernatant was precipitated with isopropanol and washed twice with 70% ethanol. DNA quantity and purity were measured using the Nanodrop ND1000. Samples with 260/280 nm absorption ratios less than 1.8 were purified again.

### SNP genotyping

We used Illumina's GoldenGate Genotyping assay, on the Illumina BeadXpress. The marker set consisted of 384 SNPs [14] ("mallard 384 SNP set"), which are numbered according to their dbSNP accession numbers from ss263068950 (SNP 0) to ss263069333 (SNP 383). Raw genotyping results were analysed in GenomeStudio (Illumina), and SNP clusters adjusted by hand. The respective OPA (oligo pooled assay) and cluster files can be found online with this paper (Additional file 7 and Additional file 8).

### SNP set evaluation

We assessed technical and biological properties of the SNP set in *Anas platyrhynchos*:

#### i) Minor allele frequencies and heterozygosity

For each of the nine localities we counted the occurrences of each of the two alleles. The count of the allele occurring less frequently (minor allele) was divided by the total number of alleles, giving the population wide frequency of minor alleles per locus (minor allele frequency, MAF). Additionally, we counted heterozygote individuals as a fraction of all individuals (observed heterozygosity, H_obs_).

#### ii) Hardy-Weinberg equilibrium

Each locus was tested for deviation from Hardy-Weinberg equilibrium in each locality with the software Arlequin 3.5.1.2 [65] using the analog to Fisher's exact test for arbitrary table size [66] (1,000,000 Markov chain steps, 100,000 dememorisation steps).

#### iii) Linkage disequilibrium

Per locality, pairs of SNP loci were tested for presence of linkage disequilibrium (LD) in Arlequin. The implemented likelihood-ratio test [67] employs the EM algorithm [68] to infer haplotypes from unphased genotypic data to test for statistical significance of LD. Repeated use of a SNP in multiple statistical tests requires a correction of the significance level α. In our 364 SNP data set each SNP is involved in 66066 pairwise tests, significance levels for LD are thus Bonferroni corrected.

#### iv) Physical SNP locations inferred from chicken genome

We searched the SNP positions on the chicken genome (from Kraus *et al. *[14]) in Ensembl [69] for chicken gene information using Bioconductor [70] with biomaRt in R [71]. We recorded if a SNP was situated in a gene, or even intron.

### Persistence times of SNPs

The equation for mean persistence time t(p) is a combination of the time to loss and to fixation [72,73]. It can be written as -4*N_e_*[(1-*p*) ln(1-*p*) + *p*ln(*p*)] where p denotes the initial MAF and N_e _the effective population size (for derivation see ref. [23], page 112, eqn. 3.10). To calculate the persistence time t(p) of a SNP, an estimate of the effective population size (N_e_) from the census population size (N_c_) is thus required. Estimates of current census population sizes (N_c_) of the investigated duck species were taken from the BirdLife species fact sheets [74]. Upper estimates were used when a range was given. The ratio between N_e _and N_c _for species of dabbling ducks has to our knowledge not been studied, but it is probably fairly low as most census estimates are based on winter counts made several months before the breeding season starts and most mortality may occur before breeding [75]. Further, dabbling ducks are generally *r*-selected and their population sizes fluctuate greatly by swift responses to benign and detrimental conditions [76,77], with N_e _being dominated by the smallest values [23]. Estimated N_e_: N_c_ ratios from white-winged wood ducks (*Asarcornis scutulata*, formerly *Cairina scutulata*) range between 0.052 (genetic measurements) and 0.094 (demographic measurements) [78]. Thus, we use a ratio of 0.1 as a conservative estimate (on the high side) for the ratio of N_e _to N_c_.

The generation time has been set to one year for clarity. As mentioned above, many individuals do not reproduce at all, and those that do are in the vast majority of the cases first-years [75]. The actual generation time value should lie in the range between 1.1 and 1.2 years, and this has no effect on our interpretations.

### Interspecific, genetic admixture

A Bayesian genetic clustering algorithm as implemented in the software STRUCTURE [37] (version 2.3.3) was used to test for genetic admixture, i.e., the incorporation of genes from one discrete population/species into another. Two datasets were analysed: i) all *Anas platyrhynchos *and other duck species (the same individuals as analysed by PCA, see Figure 2c); ii) only the other species (*cf*. Figure 2b) plus the putative hybrid between *Anas platyrhynchos *and *Anas acuta*. A value of K = 6 simulated clusters (as many clusters as species) was chosen in the analysis of all ducks (i), and consequently K = 5 when *Anas platyrhynchos *was excluded (ii). Default settings were used with the admixture model of STRUCTURE, run for 300,000 steps (the first 100,000 discarded as burn-in). Additionally, we compared the results of the STRUCTURE analysis with those of the program InStruct [38] which is designed to perform the same analysis as STRUCTURE but not depending on Hardy-Weinberg equilibrium. The same datasets and settings were used, including the default settings, with the same values for K. Mode 1 - "infer population structure only with admixture" - in InStruct was chosen because it is most comparable to the program STRUCTURE as explained in its manual. The dataset containing only non-*Anas platyrhynchos *ducks (K = 5) was also run for the same amount of iteration steps. The larger dataset, all ducks combined (K = 6), was run substantially longer because the Markov chain converged very slowly (2,000,000 steps, of which 1,000,000 were discarded as burn-in).

### Multivariate genetic clustering of genotypes

We tested for genetic similarity of individuals using principal component analysis (PCA) on their genotypes with the program smartpca from the Eigenstrat package [79] with default settings, but outlier removal switched off. The analysis was repeated for every new subset of the data.

## Competing interests

The authors declare that they have no competing interests.

## Authors' contributions

RHSK designed the study, coordinated sample collection, prepared DNA, analysed and interpreted data, and wrote the manuscript. PvH analysed and interpreted the data, and revised the manuscript. HHDK analysed data. H-JM interpreted data and revised the manuscript. RCY interpreted data, co-wrote the manuscript, and coordinated sample collection. JE co-wrote the manuscript. RPMAC, MAMG, AT and HHTP revised the manuscript. AT, DS and SAS coordinated sample collection and discussed the paper. All authors read and approved this paper.

## Supplementary Material

Additional file 1 **Bar graph of the genetic admixture analysis of individual ducks**. All duck species in one admixture analysis. Each bar represents one individual and colours indicate membership in a certain cluster as identified with STRUCTURE without using prior information. Individual IDs are explained in the text and additional file 4 and additional file 6. On the y-axis the percentage of membership in a certain cluster is given. For instance, individual ATHO001 (individual 1 from the *Anas platyrhynchos *locality in Austria) is almost 100% assigned to the light blue cluster, while individual CARM009 (from a Canadian locality) is mainly assigned to the light blue, but also with about 15% to the purple cluster (an effect of genetic admixture between these two otherwise discrete clusters). This file is scalable in order to retrieve details if needed. Click here for file

Additional file 2 **Bar graph of the genetic admixture analysis of individuals: *Anas platyrhynchos *excluded**. See additional file 1 for details. Click here for file

Additional file 3 **Vector graph of a PCA analysis of genotypes of all duck species**. First and second axes are plotted against each other (explained variation in brackets). Grey dots represent individuals designated as *Anas platyrhynchos *at sampling. Other colours indicate other duck species. A tentative hybrid between *Anas acuta *and *Anas platyrhynchos *is in red. Labels next to the dots represent individual study IDs. This file is scalable in order to retrieve details if needed. Click here for file

Additional file 4 **List of all *Anas platyrhynchos *samples analysed in this study**. Includes information on specific ID, collection date, country of origin, names of collectors, sampling locations, and further additional info. Click here for file

Additional file 5 **PCA analysis of genotypes of all non-*Anas platyrhynchos *individuals**. Details as in additional file 3, but without *Anas platyrhynchos*. Click here for file

Additional file 6 **List of all other duck species samples analysed in this study, Details similar those given in **additional file 4. Click here for file

Additional file 7 **'Oligo pooled assay (OPA)' summary file**. Contains all necessary information for genotyping the presented SNPs on an Illumina BeadXpress system. Click here for file

Additional file 8 **'Cluster file' for use with GenomeStudio software**. These configuration settings are used by the SNP genotyping software to convert raw signal into genotypes. Click here for file
