# Supplementary figures and images for: Widespread horizontal genomic exchange does not erode species barriers among sympatric ducks
Source: BMC Evol Biol. 2012 Apr 2;12:45. doi: 10.1186/1471-2148-12-45 (PMC3364866; doi:10.1186/1471-2148-12-45)

# PCA all

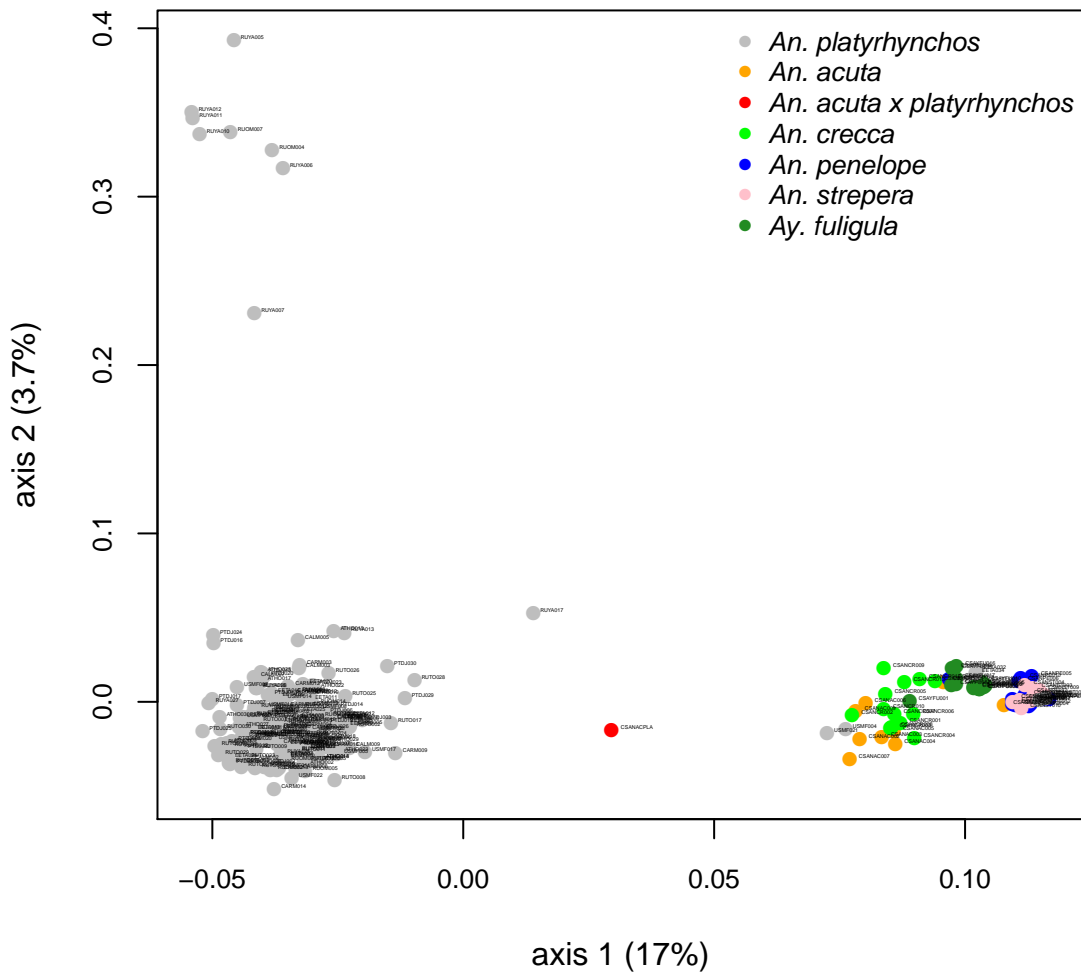

Supplement: Additional file 1 — Bar graph of the genetic admixture analysis of individual ducks. All duck species in one admixture analysis. Each bar represents one individual and colours indicate membership in a certain cluster as identified with STRUCTURE without using prior information. Individual IDs are explained in the text and additional file 4 and additional file 6. On the y-axis the percentage of membership in a certain cluster is given. For instance, individual ATHO001 (individual 1 from the Anas platyrhynchos locality in Austria) is almost 100% assigned to the light blue cluster, while individual CARM009 (from a Canadian locality) is mainly assigned to the light blue, but also with about 15% to the purple cluster (an effect of genetic admixture between these two otherwise discrete clusters). This file is scalable in order to retrieve details if needed. [file 1471-2148-12-45-S1.PDF]

## PCA non-mallards

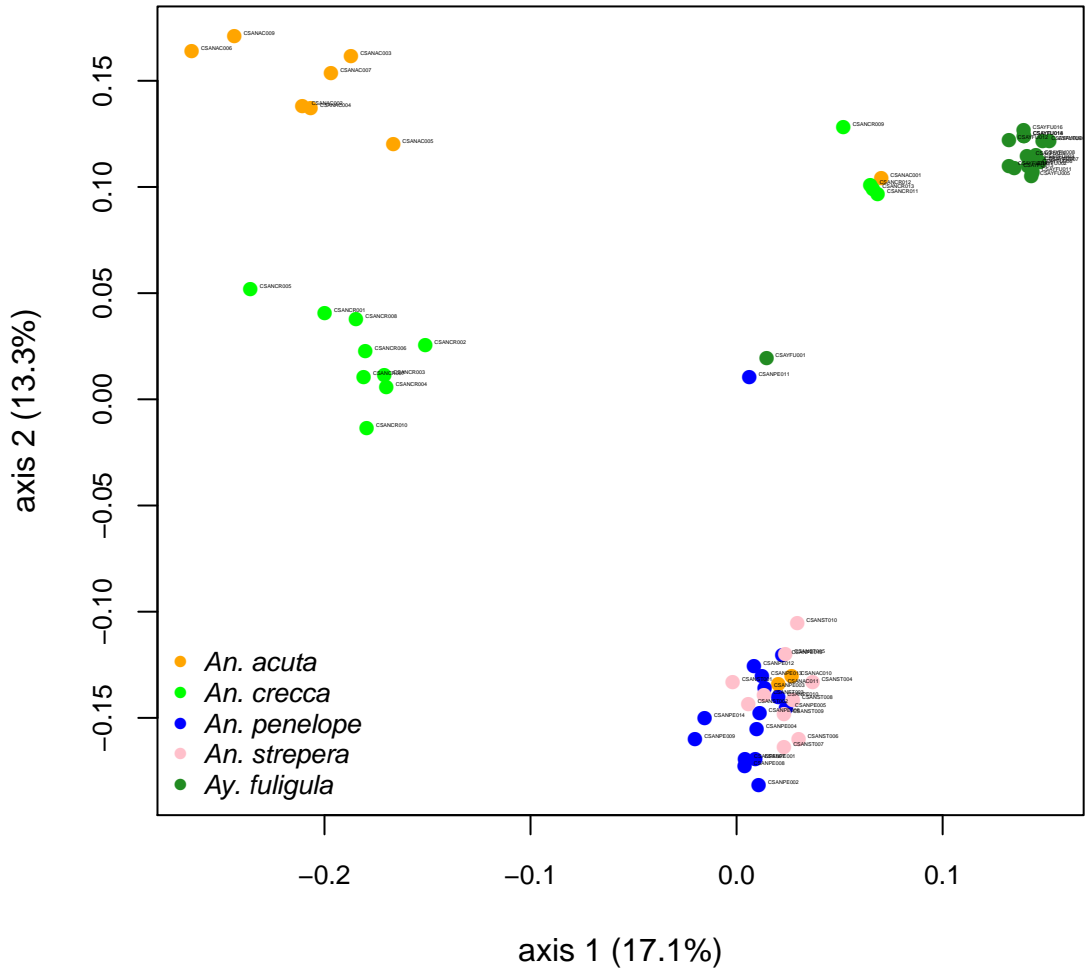

Supplement: Additional file 2 — Bar graph of the genetic admixture analysis of individuals: Anas platyrhynchos excluded. See additional file 1 for details. [file 1471-2148-12-45-S2.PDF]

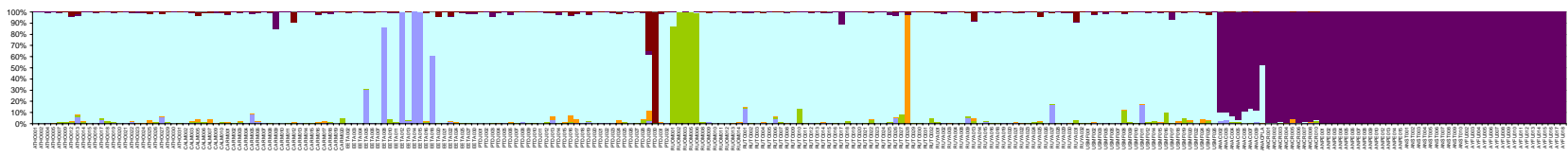

Supplement: Additional file 3 — Vector graph of a PCA analysis of genotypes of all duck species. First and second axes are plotted against each other (explained variation in brackets). Grey dots represent individuals designated as Anas platyrhynchos at sampling. Other colours indicate other duck species. A tentative hybrid between Anas acuta and Anas platyrhynchos is in red. Labels next to the dots represent individual study IDs. This file is scalable in order to retrieve details if needed. [file 1471-2148-12-45-S3.PDF]

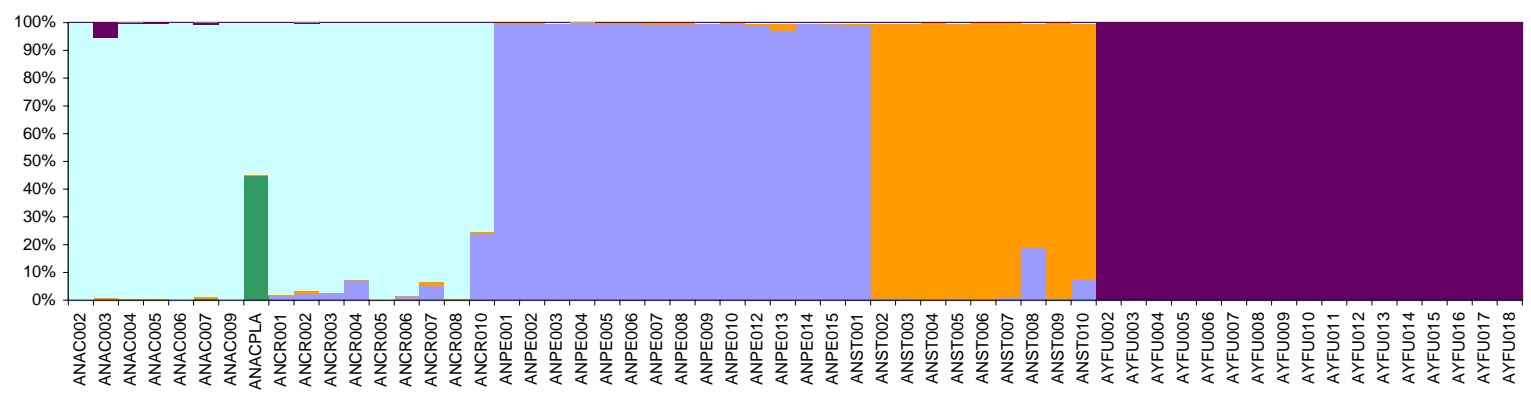

Supplement: Additional file 4 — List of all Anas platyrhynchos samples analysed in this study. Includes information on specific ID, collection date, country of origin, names of collectors, sampling locations, and further additional info. [file 1471-2148-12-45-S4.PDF]
